# An Explainable AI-Based Fault Diagnosis Model for Bearings

**DOI:** 10.3390/s21124070

**Published:** 2021-06-13

**Authors:** Md Junayed Hasan, Muhammad Sohaib, Jong-Myon Kim

**Affiliations:** 1 Department of Electrical, Electronics and Computer Engineering, University of Ulsan, Ulsan 44610, Korea; junhasan@gmail.com; 2 Department of Computer Science, Lahore Garrison University, Lahore 54000, Pakistan; md.sohaibdurrani@gmail.com

**Keywords:** bearing, Boruta, condition-based monitoring, explainable AI, fault diagnosis, model interpretability, SHAP, Stockwell transform

## Abstract

In this paper, an explainable AI-based fault diagnosis model for bearings is proposed with five stages, i.e., (1) a data preprocessing method based on the Stockwell Transformation Coefficient (STC) is proposed to analyze the vibration signals for variable speed and load conditions, (2) a statistical feature extraction method is introduced to capture the significance from the invariant pattern of the analyzed data by STC, (3) an explainable feature selection process is proposed by introducing a wrapper-based feature selector—Boruta, (4) a feature filtration method is considered on the top of the feature selector to avoid the multicollinearity problem, and finally, (5) an additive Shapley explanation followed by k-NN is proposed to diagnose and to explain the individual decision of the k-NN classifier for debugging the performance of the diagnosis model. Thus, the idea of explainability is introduced for the first time in the field of bearing fault diagnosis in two steps: (a) incorporating explainability to the feature selection process, and (b) interpretation of the classifier performance with respect to the selected features. The effectiveness of the proposed model is demonstrated on two different datasets obtained from separate bearing testbeds. Lastly, an assessment of several state-of-the-art fault diagnosis algorithms in rotating machinery is included.

## 1. Introduction

Rotating machinery has become a vital part of modern industry [1,2]. Due to their regular use and the erratic working environment of industries, maintenance of these machineries is inevitable [3]. The rolling element bearing is one of the essential components of these machines. It is the most critical component because it is highly susceptible to damage due to various factors such as variable speed and load, multiple fault severities, and ample noise alternative load conditions [4]. As these components are likely to experience frequent wear and tear, they become the primary reason for the sudden failures of the rotating machineries [1]. In return, industry may face unexpected downtime, huge economic loss, and safety related issues [5,6,7,8]. Thus, in the past few decades, industries have recognized the significance of reliable and robust Condition Monitoring and Fault Diagnosis (CM-FD) techniques to mitigate these issues [9]. These CM-FD techniques can be developed using data from different modalities, including acoustic emissions [10], vibration acceleration signals [11], ultrasonic signals [12], and signature analysis of the motor current signals [7]. Among them, vibration signals are a popular choice for the bearing CM-FDs because these signals contain clear fault-related signatures and can be explored easily through signal processing techniques [13,14]. For the past several years, researchers have been trying to develop a generalized approach for bearing CM-FD that can identify faults from the given data with high efficacy. These approaches usually focus on two parts, i.e., (a) to identify the fault pattern from the extracted features by various signal processing algorithms, and (b) by utilizing those distinguishable patterns to develop a classification/prediction algorithm using different Machine Learning (ML) or Deep Learning (DL)-based approaches.

Throughout the past few years, researchers have relied on several signal processing techniques, such as Fast Fourier Transformation (FFT) [15], Empirical Mode Decomposition (EMD) [16], Energy Entropy (EE) [17], Wavelet Packet Decomposition (WPD) [18], Empirical Wavelet Transformation (EWT) [19], Variational Mode Decomposition (VMD) [20], and Continuous Wavelet Transform (CWT) [21], as an integral part of the bearing CM-FD pipeline. One of these works is by Shao et al. [15], which proposes a bearing fault diagnostic framework that analyzes the vibration signals with FFT with a Deep Boltzmann Machine (DBM). Similarly, in [22], Zheng et al. introduced a multiscale fuzzy entropy-based architecture to measure the time-series complexity with the sequence of Laplacian Score (LS), and introduced a variable predictive model to create the diagnostic framework. Ali et al. proposed a feature extraction procedure by analyzing the Empirical Mode (EM) and the EE from vibration signals, and used an Artificial Neural Network (ANN) to develop the diagnostic framework in [17]. Furthermore, Sun et al. [16] designed a fault diagnosis model for bearings by using EMD-based imaging with an improved Chebyshev distance. Likewise, in [21], Cheng et al. used a CWT-based local binary pattern formation technique to develop the diagnosis framework by a Convolutional Neural Network (CNN). By introducing a WPD-based multi-scale permutation entropy with a Hidden Markov Model (HMV), Zhao et al. proposed a diagnostic framework for bearings in [18]. Likewise, in [19], Zhicheng et al. proposed an improved version of the Empirical Wavelet Transformation (EWT) technique to capture the fault information from noisy vibration signals, and used a Support Vector Machine (SVM) to complete the task of diagnosis. Very recently, an adaptive VMD with a Teager Energy Operator (TEO) for diagnosing the incipient fault of rolling bearings was introduced by Gu et al. [20]. All these approaches use some signal processing techniques to extract fault-related information from the signals. Apart from these signal-based analysis techniques, several model-based theoretical approaches were studied by numerous existing literatures. In [23], Immovilli et al. conducted a theoretical analysis of the physical link between faults for vibration and motor current signal, formed as a torque disturbance, and current components. Thus, they proposed a theoretical development of the correlation between torque disturbances and the amplitude of the current components. In [24], by analyzing the suitable position of the flux probe for a wide range of power levels, a fault detection approach is designed based on the stray flux around the motor for ball bearing. Likewise, in [25], Frosini et al. examined the possibility to employ a simple external flux coil to monitor the operating conditions of induction motors of different sizes. These approaches also provide satisfactory performance under constant speed conditions of rotary machines with variable locations of the flux probes. However, due to several factors, i.e., friction among components of a machine, misalignment, and noise, the vibration signals acquired from bearings are non-linear and non-stationary in nature, which creates difficulties when extracting and analyzing the fault feature information from different speed conditions [3,11,26,27]. Specifically, via the popular feature extraction methods which analyze features from the time domain, frequency domain, or time-frequency domain, it becomes very difficult to identify the fault characteristics for variable working conditions [10,28,29]. To analyze such complex signals, these signal processing techniques suffer some limitations given as follow:

(1) In practice, bearing signals are prone to inconsistencies due to several factors which make them difficult to analyze. Therefore, if there is a little variation in the given CM-FD scenario, the adopted signal processing techniques are unable to extract fault characteristics properly.
(2) Wavelets transform-based signal analysis usually experiences the dilemma of appropriate Mother Wavelet (MW) selection and suffer from the drawbacks of poor noise immunity.

Therefore, it is inevitable to come up with a new and effective signal processing technique through which fault signature exploration can become reliable during the CM-FD of bearings [30,31]. Recently, in this regard, Stockwell Transform (ST) was used for the fault signature identification and localization [32] for the following major advantages:

(1) It has better immunity to ample noise.
(2) It is free from the MW selection.
(3) It can obtain good resolutions from the signals both at low and high frequencies. Thus, it has a built-in adaptive ability to tackle inconsistencies in the observed data.

However, due to the redundant nature for selecting Gaussian windows, ST suffers from a computational complexity of $O\left(N^{2}\log N\right)$. Therefore, in this study, a non-redundant faster variant of ST, known as the Faster Discrete Orthogonal Stockwell Transform (FDOST) with a computational complexity of $O\left(N\log N\right)$, is proposed to explore unique patterns for a given fault. In this regard, first, the FDOST coefficients are computed from the vibration signals which are then utilized to extract statistical parameters from both time-frequency magnitudes and their corresponding phase angle information.

After the application of signal processing techniques, the CM-FD pipeline normally utilizes feature extraction and a selection step. Generally, first, the statistical features are extracted from the processed signals, and afterwards, useful features are selected out of the whole feature pool through feature selection techniques that contain discriminant information for different fault types. Likewise, a suitable 2D representation of the selected features is an appropriate choice when dealing with a DL algorithm to accomplish the classification task. However, these approaches lack an appropriate explanation of the feature selection process. Additionally, they are unable to explain the importance of each feature in the selected feature subset, and the justification about their influence on the final output of the CM-FD pipeline is also missing. On the other hand, the ML-based approaches adapted in the field of bearing fault diagnosis completely suffer for the lack of feature and model explainability. Thus, without a clear interpretation of the model learning and working processes, it difficult to debug the outcome of these diagnosis models. Moreover, these models are also not generalized, i.e., whenever any of the underlying conditions changes regarding the CM-FD process, changes are required in the suggested CM-FD approach to address the changes. This problem can be explained further by the subsequent points:

(1) The feature selectors available in the literature of bearing fault diagnosis are either the evolutionary algorithm-based approach [33,34] or the filter-based approach [35]. The evolutionary algorithms (i.e., genetic algorithms) select features based on the grid search and knap-snack mechanism [36] with gene cross-over and mutation. Thus, the reason for selecting an individual feature attribute can never be explained or tracked down. In addition, both approaches rely on the technique of removing the redundant feature attributes from the original feature set. Moreover, several data compression techniques, i.e., principal component analysis [37], and different manifold learning techniques are also used to represent the data into lower dimension for diagnosis purpose [38]. However, these techniques are based on algebraic calculation and geometric projection to create the separability into the feature space. Therefore, explainability remains the problem for these linear and non-linear dimensionality reduction-based techniques.
(2) Without a proper understanding of the feature selection process, all the ML-based classifiers only focus on the classification accuracy, like a black box approach. This creates an adaptability issue for the model when the working environment changes.

In this study, we have considered an Explainable Artificial Intelligence (XAI) model for bearing fault diagnosis which consists of a wrapper-based approach named Boruta, a Spearman’s Rank Correlation Coefficient (SRCC), and a k-NN classifier with Shapley additive explanations. Boruta is built around a non-parametric classifier—Random Forest (RF). It first shuffles and permutes all the original feature attributes to create a randomized copy of the original feature set. Then, this randomized set of features is added to the original feature set to perform the feature selection process by an RF classifier [39]. Thus, by adding randomness to the feature attributes, this algorithm outperforms the conventional forward/backward selection-based wrapper approaches [40]. Subsequently, with the help of an embedded RF classifier, the selection of every feature attribute can be explained. Before feeding the selected features to the classifier, we have proposed an additional collinearity-based feature filtration step by using a Spearman’s Rank Correlation Coefficient (SRCC) [41,42] to obtain a bias-free accuracy measurement. This SRCC helps to remove the correlated feature attributes from the extracted statistical feature attributes. Thus, the explanations for selecting feature attributes give the opportunity to design a biasfree explainable ML model. To decide the best classifier, we need to adapt a simple, yet powerful model which does not get affected by the heteroscedasticity [43] of the data. To satisfy all these conditions, in this study, we have considered k-Nearest Neighbor (k-NN) as a classifier. Finally, to interpret the decision of k-NN, we have proposed the kernel Shapley Additive Explanation (SHAP) [44] into our diagnosis model. There are two main advantages of this SHAP-based model explanation.

(1) This interpretation of the Shapley value is inspired from a collaborative game theory scenario where contributions of each feature attribute on the model performance are unequal but in cooperation with each other [45]. The Shapley value guarantees each feature attribute profits as much or more as they would have from performing independently.
(2) The SHAP kernel can provide a unique solution by satisfying local accuracy, missingness, and consistency on the basis of the original model, hence allows explainability of a model [44]. The proof of these properties will be discussed later in Section 2.5 of this manuscript.

Furthermore, the contributions of the proposed XAI model for bearing fault diagnosis can be summarized as follows:

(1) To capture the information of variable working conditions from the vibration signals both at low and high frequencies, a computationally advanced version of ST called FDOST is proposed as the signal preprocessing step. First, statistical parameters are extracted as features from the time-frequency magnitudes and their corresponding phase angles of the FDOST coefficients. The extracted statistical features are then arranged as a feature matrix which can be regarded as input in the proceedings step. Thus, a carefully curated statistical feature pool extracted from unique FDOST patterns for different types of bearing faults is proposed in this study, which is helpful for boosting up the classification performance of the subsequent classifier.
(2) A wrapper-based feature selector named Boruta is utilized to find the best features from the extracted statistical feature pool. This algorithm can justify the selection of each feature attribute with the help of an embedded RF classifier. Thus, the feature selection process is easily interpretable in the proposed bearing fault diagnosis model.
(3) In addition to interpretable feature selection process, a feature filtration technique is proposed by using SRCC to create a reduced feature set for the classifier which can produce bias free results. Thus, it helps the classifier to avoid the multicollinearity trap.
(4) A correlation between the filtered feature pool and results of a non-parametric k-NN classifier is presented in this work, i.e., the predictions of k-NN are explained in context of SHAP values.

This work introduces the concept of explainability for the first time in the field of bearing fault diagnosis in two steps: (a) incorporating explainability of the feature selection process, and (b) interpretation of the ML classifier performance with respect to the selected features. Thus, it makes the proposed model a state-of-the-art XAI-based fault diagnosis approach for bearings, which is applicable in real-world scenarios.

To validate the proposed model, two bearing datasets have been considered, among which, one is obtained from the public repository of Case Western Reserve University [46], and the other one is collected from a self-designed test bed. The improvement in the performance of the proposed signal processing step, feature selection process, and classifier has been verified through several comparisons with state-of-the-art published studies. The further details related to the dataset description along with the experimental setup are discussed in Section 4.

The complete organization of this manuscript can be summarized as follows: Section 2 gives the theoretical and mathematical descriptions of the necessary backgrounds, Section 3 discusses the proposed methodology in a step-by-step procedure, Section 4 describes the description of the datasets, Section 5 highlights the experimental analysis with discussion, and Section 6 concludes this work.

## 2. Technical Background

This section discusses the technical details of the Fast Discrete Orthogonal Stockwell Transformation (FDOST) wrapper-based feature selector—Boruta, the Spearman’s Rank Correlation Coefficient (SRCC), the k-Nearest Neighbor (k-NN) algorithm, and the Shapley Additive Explanation (SHAP) for model interpretation.

### 2.1. Fast Discrete Orthogonal Stockwell Transformation

The Stockwell Transformation (ST) is a multi-resolution evaluation-centered method which delivers a frequency domain transformation like the Fourier Transform (FT) [47], introduced by R.G Stockwell [48]. Initially it was defined to capture good resolution from signals at both low frequencies (wide window size) and high frequencies (small window size), like the Continuous Wavelet Transform (CWT) with a Gaussian window [49]. Moreover, it can also provide phase information associated with a signal, where CWT fails. The ST spectrum provides local phase information which is helpful in defining distinct clusters for different classes [50]. However, the initially proposed algorithm with the Gaussian window had redundancy and created a high computational cost $O\left(N^{2}\log N\right)$ [50]. Later, a significant number of studies helped to resolve this issue [51]. In this study, one of the most efficient variations of the ST proposed by U. Battisti et al. [52] is considered. This version of the algorithm offers a unified setting with a different admissible window to reduce the computational complexity to $O\left(N\log N\right)$, while calculating the ST coefficients [52].

The ST of a function can be defined as:

(1) $$S\left(\tau ,f\right)=\overset{+\infty }{\underset{-\infty }{\int }}h\left(t\right)\frac{\left|f\right|}{\sqrt{2\pi }}\exp\left(-\frac{{\left(\tau -t\right)}^{2}}{2f^{2}}\right)\exp\left(-i2\pi ft\right)dt$$

where f is the frequency, t and τ are the time variables, and $\left|f\right|/\sqrt{2\pi }$ is the normalizing coefficient factor. This continuous representation of the ST can be represented in a discrete form by the following equation:

(2) $$S\left[j,n\right]=\overset{N-1}{\underset{m=0}{\sum }}H\left(m+n\right)\exp\left(-\frac{2\pi^{2}m^{2}}{n^{2}}\right)\exp\left(i\frac{2\pi mj}{N}\right)\;\mathrm{for}\;n\neq 0$$

Here, to calculate the discrete ST (DST), the following equivalence parameters are considered, i.e., ∫→∑,f→n, and τ→j. In Equation (2), $H\left[.\right]$ is the Discrete Fourier Transform (DFT) of $h\left[.\right]$, which is the FT in Equation (1). Equation (2) can be simplified as:

(3) $$S\left[j,n\right]=\frac{1}{N}\overset{N-1}{\underset{m=0}{\sum }}h\left[k\right]\;\mathrm{where}\;k=0,T,2T,\ldots ,\left(N-1\right)T$$

where $h\left[k\right]$ is the discrete representation of $h\left(t\right)$. However, Equation (2) can generate an $N^{2}$ number of coefficients for a signal of length N. Thus, it creates a computational complexity of $O\left(N^{3}\right)$. This high redundancy can be reduced by introducing N number of orthogonal basis vectors for the calculation of the number of coefficients for the ST. This approach is established as the Discrete Orthogonal Stockwell Transform (DOST). Mainly by the values of the three parameters, i.e., ν (center of a frequency band), β (width of the frequency band), and τ (location in time), the $k^{th}$ basis vectors of the DOST can be represented as follows:

(4) $$D{\left[k\right]}_{\left[\nu ,\beta ,\tau \right]}=\frac{1}{\sqrt{\beta }}\overset{\nu +\beta /2-1}{\underset{f=\nu -\beta /2}{\sum }}\exp\left(i\frac{2\pi kf}{N}\right)\exp\left(-i\frac{2\pi \tau f}{\beta }\right)\exp\left(-i\pi \tau \right)$$

Therefore, the DOST coefficient can be calculated as the inner product of the basis vector $D{\left[k\right]}_{\left[\nu ,\beta ,\tau \right]}$ and the input signal $h\left(k\right)$.

(5) $$S_{\left[\nu ,\beta ,\tau \right]}=\langle D{\left[k\right]}_{\left[\nu ,\beta ,\tau \right]},h\left(k\right)\rangle \;\mathrm{where}\;k=0,1,2\ldots ,\left(N-1\right)$$

In Equation (5), the DOST coefficients are decreased to N with a computational complexity of $O\left(N^{2}\right)$. However, to reduce it further, a faster technique is proposed by using the advantage of the Fast Fourier Transform (FFT) in [51]. This approach reduced the computational complexity to $O\left(N\log N\right)$ by using a fixed window size. Therefore, to introduce this Faster Discrete Orthogonal Stockwell Transform (FDOST), the coefficients are derived from Equation (5) as follows:

(6) $$S_{\left[\nu ,\beta ,\tau \right]}=\frac{1}{\sqrt{\beta }}\overset{N-1}{\underset{k=0}{\sum }}\overset{v+\beta /2-1}{\underset{f=v-\beta /2}{\sum }}\overset{\nu +\beta /2-1}{\underset{f=\nu -\beta /2}{\sum }}\exp\left(i\frac{2\pi kf}{N}\right)\exp\left(-i\frac{2\pi \tau f}{\beta }\right)\exp\left(-i\pi \tau \right)h\left(k\right)\;=\frac{1}{\sqrt{\beta }}\overset{\nu +\beta /2-1}{\underset{f=\nu -\beta /2}{\sum }}\exp\left(-i\pi \tau \right)\exp\left(-i\frac{2\pi \tau f}{\beta }\right)H\left[f\right]$$

where $H\left[f\right]$ is the DFT of $h\left[k\right]$. To improve this representation, U. Battisti et al. [52] proposed a generalized window (φ) dependent basis function which can be defined as:

(7) $$E_{\left[p,j\right]}^{\varphi }\left(k\right)=\frac{1}{\sqrt{\beta \left(p\right)}}{\overset{\beta \left(p\right)-1}{\underset{j=0}{\sum }}\left[C_{\left[p,j\right]}^{\varphi }\left(\nu \left(p\right)\right)\right]}^{-1}\exp\left(2\pi i\left(\beta \left(p\right)+j\right)\left(\frac{k}{N}-\frac{\tau }{\beta \left(p\right)}\right)\right)$$

where *p* denotes the frequency bands determined by ν and β. The coefficient of the FDOST can be calculated as:

(8) $$S_{\left[p,\tau \right]}^{\varphi }=\langle h\left(k\right),E_{\left[p,\tau \right]}^{\varphi }\rangle =\langle g,D{\left[k\right]}_{\left[p,\tau \right]}\rangle =\langle F^{-1}R^{\varphi }H,D{\left[k\right]}_{\left[p,\tau \right]}\rangle$$

where $F^{-1}$ is the Inverse Fourier Transform (IFT), H is the DFT of $h\left(k\right)$, $R^{\varphi }$ is the sequence function, and $D{\left[k\right]}_{\left[p,\tau \right]}$ is the modified basis vector for calculating the coefficient of the FDOST. Therefore, the updated Equation (4) for calculating the $k^{th}$ basis vectors of the FDOST with a generalized window can be represented as follows:

(9) $$D{\left[k\right]}_{\left[p,\tau \right]}=\frac{1}{\sqrt{\beta \left(p\right)}}\overset{\beta \left(p\right)-1}{\underset{j=0}{\sum }}\exp\left(i2\pi \left(\beta \left(p\right)+j\right)\frac{k}{N}\right)\exp\left(-i\frac{2\pi \tau j}{\beta \left(p\right)}\right)$$

**Figure 1:**
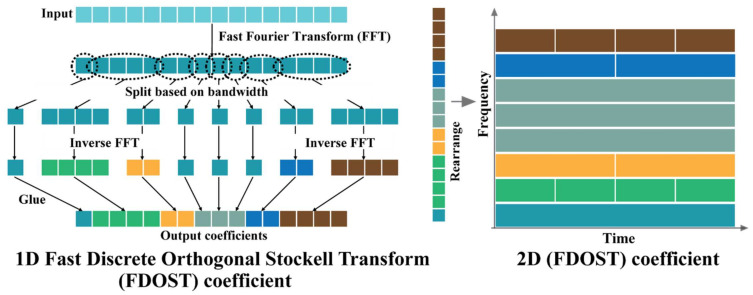
A visualization of the FDOST coefficient calculation process.

The process of calculating the FDOST coefficient is illustrated in Figure 1 for a visual understanding.

### 2.2. Wrapper-Based Feature Selector—Boruta

A wrapper approach usually uses a non-parametric classifier for the selection of important features from the complete feature pool. The reason behind considering a non-parametric classifier is to reduce the computational complexity [53], along with avoiding the multicollinearity problem [54]. Boruta is a wrapper-based approach that uses the Random Forest (RF) classifier for the feature selection process. This algorithm first shuffles and permutes all the original feature attributes to create a randomized copy of original feature set. Then, this randomized set of features is added to the original feature set to bring randomness to the feature attributes. Finally, it performs the feature selection process by the RF classifier on those attributes [35]. Thus, by adding randomness to the feature attributes, this algorithm outperforms the conventional forward/backward selection-based wrapper approaches [39,40]. The steps of the Boruta algorithm are described below:

(1) By shuffling and permuting all the original feature attributes, it first creates a randomized copy of the original feature set. These replicated feature sets are known as Shadow Attributes (SAs). Then, these SAs are merged with the original feature attributes to form the Extended Information System (EIS) [40]. At least five SAs are required to form the EIS.
(2) Then, the values of these SAs are randomly permuted and shuffled. Thus, pure randomness is present in all the replicated variables and the decision attributes.
(3) Afterwards, an RF classifier is fitted to the EIS several times, and the SAs are randomized before each run. Therefore, for each run, the randomly updated part of the EIS is distinct.
(4) For each run, the importance of all feature attributes (Z score) is computed. To compute this importance, the following steps are considered:
  (a) The EIS is divided into several Bootstrapped Sets of Samples (BSSs) (in another word, the training samples) equivalent to the considered number of Decision Trees (DTs) used for the RF. Therefore, the samples for testing, which are commonly known as the Out of the Bag Samples (OOBSs), are equivalent to the number of BSSs.
  (b) Then, each BSS is used for training individual DTs, whereas the corresponding OOB is used for testing the performance of that DT. Thus, for each feature attributes from the EIS, the number of votes for the correct class are recorded.
  (c) Later, the values of the feature attributes of the OOBs are randomly permuted to record the votes for the correct class once more, like the previous step.
  (d) Then, the importance of the values of the attribute for a single DT can be calculated as follows:
    (10) $$MDA=CorrectVotesCast_{original}-CorrectVotesCast_{permuted}$$
  This importance measure is known as the Mean Decrease in Accuracy (MDA).
  (e) Finally, the importance of the values of the feature attribute ($V_{i}$) throughout the forest is computed as follows:
    (11) $$V_{i}=\frac{1}{N}\left(\overset{N}{\underset{n=1}{\sum }}MDA_{n}\right)\;\mathrm{where}\;N\;\mathrm{indicates}\;\mathrm{the}\;\mathrm{total}\;\mathrm{number}\;\mathrm{of}\;\mathrm{DT}.$$
  (f) Therefore, the final importance score is calculated as:
    (12) $$Z=\frac{V_{i}}{\sigma_{V_{i}}}$$
(5) Find the Maximum Value of Z among the Shadow Attributes (MVSA). After that, assign a hit to every attribute that scored higher than the MVSA. To determine the best attributes, the following steps are considered:
  (a) Consider an attribute as important if it performs significantly higher than the MVSA.
  (b) Remove an attribute from the EIS as non-important if it performs significantly lower than the MVSA.
  (c) For an attribute with undetermined importance, a two-sided test of equality is conducted with the MVSA.
(6) Remove all the SAs from the EIS.
(7) Repeat the whole process till any of the following two cases are satisfied:
  (a) The importance is assigned to all the attributes.
  (b) The algorithm reaches the limit of defined number of RF runs.

**Figure 2:**
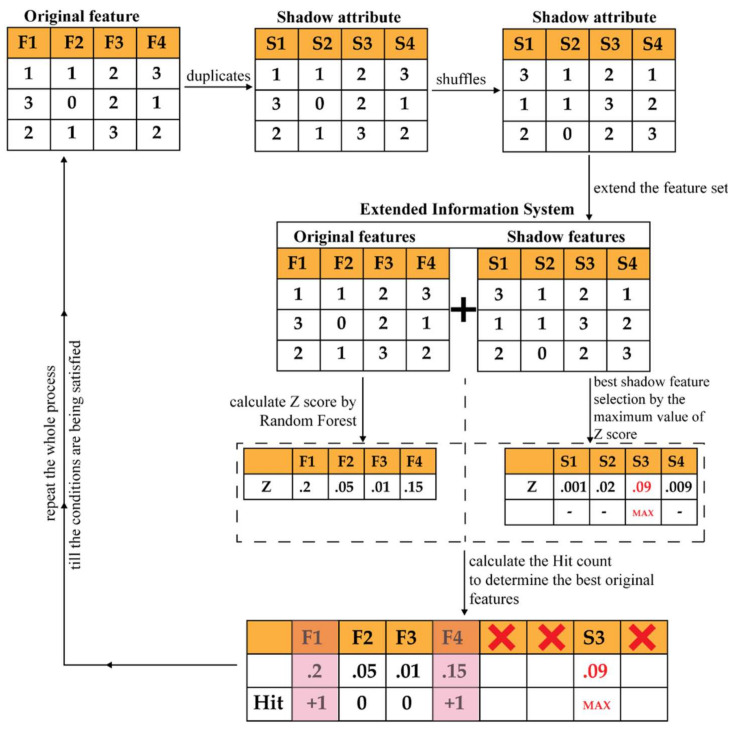
A visual representation of Boruta.

The complete process is illustrated in Figure 2.

### 2.3. Spearman’s Rank Correlation Coefficient

Correlation is a statistical relationship between two random variables in bivariate data [55]. In other words, it defines the degree of the linear relationship between two random variables. However, correlation never implies causation of the data. The Correlation Coefficient (CC) is the statistical measure used to calculate the relationship between the relative movements of two random variables. Among various CC measurement techniques [56,57], Spearman’s Rank Correlation Coefficient (SRCC) is a non-parametric test to measure the correlation [41,42]. There are two main advantages of SRCC over the rest of the CC measurement techniques:

(1) Due to the non-parametric test approach, it carries no assumptions of the distribution of the data [42].
(2) SRCC works on rank-ordered variables. Therefore, it works with the variables which have linear or monotonic relationships [42,55].

The SRCC can be calculated by the following equation,

(13) $$\rho =1-\frac{6\sum d_{i}^{2}}{n\left(n^{2}-1\right)}$$

where n represents the number of observations and $d_{i}$ is the distance between the ranks of the corresponding variables.

### 2.4. k-Nearest Neighbor Algorithm

**Figure 3:**
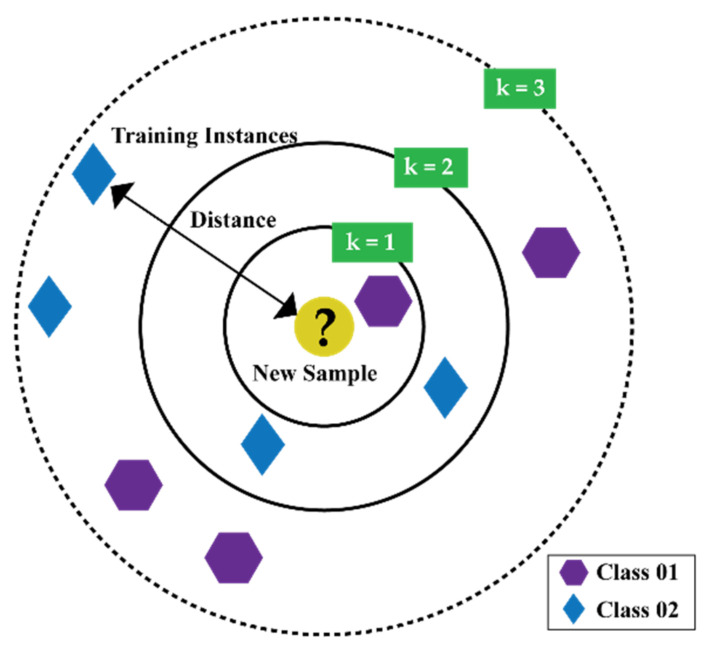
An illustration of the k-NN algorithm.

The k-Nearest Neighbor Algorithm (k-NN) is one of the simplest algorithms of ML [58,59]. This algorithm starts by assuming that similar things are close to each other, as described in Figure 3. Utilizing the idea of similarity based on proximity or distance, k-NN calculates the distance between two points by different approaches, i.e., Euclidian [60], Manhattan [61]. However, in general, among all these approaches, Euclidian is the most widely used approach, which can be expressed as follows:

(14) $$d\left(m,b\right)=\sqrt{\overset{n}{\underset{i=1}{\sum }}{\left(m_{i}-b_{i}\right)}^{2}}$$

where m,b are two points in an n dimensional Euclidian space.

To determine the chosen number of k (in other words, the number of neighbors) for a certain dataset, k-NN needs to be run several times with different values of k. From each run, select the value of k which reduces the number of errors while making predictions. However, few things shall be considered while choosing the number of k.

(1) If the value of k is set to 1, the prediction turns out to be less stable.
(2) Inversely, if the value of k is increased, the prediction becomes stable due to the majority voting/averaging. However, after a certain value for the target dataset, the number of errors will start to increase.
(3) The optimal k value can be determined by considering the square root of N, where N is the total number of samples. Then, by using a grid search approach, from 1 to the optimal value of k, an error plot or accuracy plot is calculated to determine the most favorable k value.
(4) Usually, when considering the values for the range of k for the grid search, each value for k is set as an odd number, i.e., [1,3,5,7,9…,optimal−k−value]. Thus, for the scenario while majority voting is necessary to determine the prediction, the tiebreaker becomes easy.

### 2.5. Shapley Additive Explanation for Model Interpretation

The additive feature attribution method can interpret/explain the individual decision/prediction of classifier [44]. This method supports the model output as a sum of real values attributed to each input feature. Additionally, this method has a distinctive characteristic to provide a unique solution for the following three properties, i.e., (1) local accuracy, (2) missingness, and (3) consistency [44].

(1) **Local accuracy:** If x is the specific input to the original model f, then for approximating f, local accuracy requires the explanation model g to match its output with the output of f for any simplified given input $x^{\prime }$ to model g.

(15) $$f\left(x\right)=g\left(x^{\prime }\right)=\phi_{0}+\overset{M}{\underset{i=1}{\sum }}\phi_{i}{x^{\prime }}_{i}=f\left(h_{x}\left(0\right)\right)+\overset{M}{\underset{i=1}{\sum }}\phi_{i}{x^{\prime }}_{i}\;\mathrm{when}\;x=h_{x}\left(x^{\prime }\right),\;\mathrm{and}\;\phi_{i}\in \mathbb{R}$$

Here, $x=h_{x}\left(x^{\prime }\right)$ is the mapping function, M denotes to the number of simplified input features, and $\phi_{i}$ denotes the effect to each feature.

(2) **Missingness:** If any feature is missing in the original input x, that feature ${x^{\prime }}_{i}$ has no attributed impact.
  (16) $${x^{\prime }}_{i}=0\Rightarrow \varphi_{i}=0\;\mathrm{when}\;x=h_{x}\left(x^{\prime }\right)$$
(3) **Consistency:** If a model is changed in a way such that a feature brings greater impact on the model, the attribution assigned to that feature will never decrease. Let $f_{x}\left(z^{\prime }\right)=f\left(h_{x}\left(z^{\prime }\right)\right)$ and $z^{\prime }\backslash i$ imply the setting for ${z^{\prime }}_{i}=0$ where $z^{\prime }\in {\left\{0,1\right\}}^{M}$. For any two models f, and $f^{\prime }$, if
  (17) $${f^{\prime }}_{x}\left(z^{\prime }\right)-{f^{\prime }}_{x}\left(z^{\prime }\backslash i\right)\geq f_{x}\left(z^{\prime }\right)-f_{x}\left(z^{\prime }\backslash i\right)\Rightarrow \phi_{i}\left(f^{\prime },x\right)\geq \phi_{i}\left(f,x\right)$$

From the above mentioned three properties, it can be easily understood that the considered additive feature attribution model is developed based on the classical methods [62,63] of estimating the solution concept in cooperative game theory called the Shapley value [45]. Therefore, SHAP values are calculated as a unified measure of feature importance as follows:

(18) $$f_{x}\left(S\right)=f_{x}\left(h_{x}\left(z^{\prime }\right)\right)=E\left[f\left(x\right)\left|x_{S}\right.\right]\;\mathrm{where}\;S\subseteq \left(index\neq 0\right)\in z^{\prime }$$

where local methods always try to ensure

(19) $$g\left(z^{\prime }\right)\approx f\left(h_{x}\left(z^{\prime }\right)\right)\;\mathrm{when}\;z^{\prime }\approx x^{\prime }$$

Previously, by Local Interpretable Model-Agnostic Explanations (LIME) [64], the explanation of this additive feature attribution method was introduced as a linear function of binary variables:

(20) $$g\left(z^{\prime }\right)=\phi_{0}+\overset{M}{\underset{i=1}{\sum }}\phi_{i}{z^{\prime }}_{i}$$

To find the $\phi_{i}$, LIME minimized the following objective function (Loss function),

(21) $$\xi =\underset{g\in G}{\arg\min}L\left(f,g,\pi_{x^{\prime }}\right)+\Omega \left(g\right)$$

where $\pi_{x^{\prime }}$ is the local kernel to calculate the loss over the set of samples in the simplified input space, and Ω denotes the penalizing constraint for reducing the time complexity [44].

On the other hand, with the Shapley regression values [44], feature importance was calculated in the presence of multicollinearity in the data by Equation (22):

(22) $$\phi_{i}=\underset{S\subseteq Q\backslash \{i\}}{\sum }\frac{\left|S\right|!\left(M-\left|S\right|-1\right)!}{M!}\left[f_{x}\left(S\cup \{i\}\right)-f_{x}\left(S\right)\right]$$

where Q is the set of all input features. However, according to the proof of Lundberg et al. in [44], g can only satisfy the above mentioned three properties by this following equation,

(23) $$\phi_{i}=\underset{z^{\prime }\subseteq x^{\prime }}{\sum }\frac{\left|z^{\prime }\right|!\left(M-\left|z^{\prime }\right|-1\right)!}{M!}\left[f_{x}\left(z^{\prime }\right)-f_{x}\left(z^{\prime }\backslash i\right)\right]$$

In this research, we have considered the kernel SHAP [ref] approach to bring the interpretability of the model. This approach uses Linear LIME (Equations (20) and (21)) with Shapley Values (Equation (23)) to calculate the feature importance.

By using Kernel SHAP, $L\left(f,g,\pi_{x^{\prime }}\right)$ from Equation (18) can be written as:

(24) $$L\left(f,g,\pi_{x^{\prime }}\right)={\underset{z^{\prime }\in Z}{\sum }\left[f\left(h_{x}{}^{-1}\left(z^{\prime }\right)\right)-g\left(z^{\prime }\right)\right]}^{2}\left[\frac{\left(M-1\right)}{\left(M_{choose}\left|z^{\prime }\right|\right)\left|z^{\prime }\right|\left(M-\left|z^{\prime }\right|\right)}\right]$$

This approach has two main advantages [44], i.e.,

(1) It can estimate the SHAP values without considering the model type.
(2) Feature importance is calculated in the presence of multicollinearity in the data.

## 3. Proposed Method

**Figure 4:**
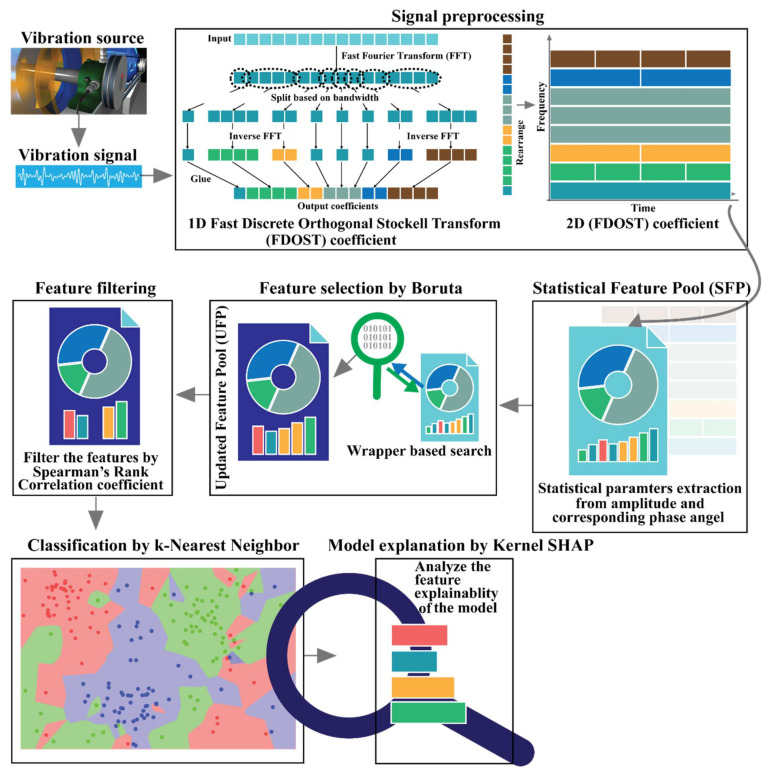
Block diagram of the proposed model for bearing fault diagnosis.

The main objective of this research is to introduce explainability to the CM-FD process of bearings, where the feature selection process and the importance of each selected feature for the ML classifier outcome can be easily interpretable. With this contribution, the generalization capability of the proposed model can be significantly improved, and it can be effectively applied to different scenarios beside those considered in this work. The block diagram of the proposed method is illustrated in Figure 4. Based on this diagram, the core steps of the proposed algorithm are described in the flowing subsections.

### 3.1. Data Preprocessing by the Fast Discrete Orthogonal Stockwell Transformation (FDOST)

The vibration signals from bearings or any rotating machineries contain the information related to the device with additive noise from the surroundings [4]. Thus, such signals are complex and possess non-stationary behavior. Therefore, it is difficult to extract the required information from these signals with traditional statistical analysis in either the time or the frequency domain [1]. To handle this issue, the FDOST has been adapted as the preprocessing step in this work. First, the raw signals are segmented into smaller sizes by adjustable overlapping sliding window [10,65]. Each of these segments contains the datapoints from at least one revolution. Then, the time domain signals are split into different bins (bandwidths) using an FFT. Next, an IFFT is performed on all the split portions except the lowest frequency bin. Afterwards, all these transformed bins are merged to form the 1D FDOST coefficient array. Finally, the 1D representations are arranged into a 2D representation. The visual explanations of the full process are already given in Figure 1. These coefficient values are complex in nature; therefore, they hold the time-frequency information along with the corresponding phase-angle information from the signals.

### 3.2. Statistical Feature Pool Configuration

After computing the FDOST of each signal, we obtain a set of complex coefficients $S_{r,c}$, which can be represented as follows:

(25) $$S_{r,c}=A_{r,c}e^{j\phi_{r,c}}$$

where *r* represents row, and *c* represents column.

Here, A and ϕ in (25) are the corresponding magnitude and phase angle of $S_{r,c}$ [66]. As previously discussed, $S_{r,c}$ contains good resolution from both low and high frequencies of the signal. Therefore, in this study, a set of statistical parameters are obtained from both the $A_{r,c}$ and $\phi_{r,c}$ matrices. Later, the maximum values are calculated from $A_{r,c}$, $A_{c,r}$, $\phi_{r,c}$, and $\phi_{c,r}$ to create four Significant Information Pools (SIPs). Thus, the maximum coefficients with respect to frequency and time for $A_{r,c}$ and $A_{c,r}$, the maximum coefficients with respect to frequency ratio, and the phase angles for $\phi_{r,c}$ and $\phi_{c,r}$ are determined from the 2D FDOST representation. Finally, from each of these SIPs, five statistical parameters (i.e., mean, standard deviation (std), kurtosis, skewness, and root-mean-square (rms)) are extracted as features. With these 20 features, for each signal, a Statistical Feature Pool (SFP) is designed for further analysis. The details of these 20 features are listed in Table 1.

### 3.3. Feature Filtering by the Spearman’s Rank Correlation Coefficient (SRCC)

In general, correlated features raise the issue of multicollinearity. Therefore, for some models, these features can adversely affect the performance due to the multicollinearity issue [67]. In this study, the considered classification algorithm is k-NN. Although it is a non-parametric approach, it can be affected because of the extra-weight carried by the correlated features on the distance calculation [68] resulting in the decline of the performance [69]. Usually, the performance discrepancy is low while dealing with a smaller feature set. Moreover, the cross-validation approach also helps to overcome the issue. However, in this study, as we have focused on the interpretability of the classifier, a simpler model is necessary with less features. This can be perceived as a special case of the Occam’s Razor principal [70,71] known as Minimum Description Length (MDL) [72,73]. Moreover, for better interpretation of the k-NN outcome with Kernel SHAP, the SRCC is chosen as a medium for dimensionality reduction of the input data over the conventional technique, i.e., Principal Component Analysis (PCA). PCA or the techniques like PCA, do not deal with collinearity, instead they just compress the original data. However, with the proposed SRCC, we can reduce the dimensionality by removing the colinear feature information from the data. Therefore, without revoking the ranking of the features by Boruta, we filtered UFP again by the SRCC. Thus, the Final Feature Pool (FFP) is obtained for the classification.

### 3.4. Classification by k-Nearest Neighbor (k-NN))

Finally, the FFP is fed to the k-NN to identify the types of faults. There are two main reasons behind selecting k-NN as a classifier in this study:

(1) It is a non-parametric clustering analysis-based model.
(2) Parameter’s tuning is not a concern for k-NN as it is a non-parametric algorithm. Therefore, assumptions about the input data are not a concern while dealing with k-NN.

### 3.5. Model Interpretation by Kernel SHAP

When a trained ML model yields an outcome for a regression or classification task, we might speculate while explaining the choice made by the model. With the accuracy-based evolution criteria of a model, we never get a complete description about the performance of a model. In the real-world cases, along with the accuracy measurement, it is necessary to understand the behavior of the model with respect to the inputs and output, so that when needed, it shall be easy to debug. Thus, with an interpretable model, we can learn more about the data and problems related to it. Therefore, the reason behind the failure of a model can be justified properly [74]. It is easy to explain a model if its decisions are easily understandable to humans. The best clarification for explaining the model is generally the model itself, since a simple model is easily represented and understood [75]. For example, the explanations of DT or RF can be easily interpreted by humans, whereas for the non-linear and complex models, it is often difficult for humans to understand the decision-making process. However, the complex models can have higher accuracy scores than the simpler models for a larger dataset. Nevertheless, the interpretability of simpler models is higher than the complex ones. Therefore, in this study, to create a sweet spot between achieving high accuracy and interpretability of the model without sacrificing any of them, we have considered k-NN as the main classifier. Due to the simple architecture of the classifier with non-parametric behavior, it is comparatively easier to explain the model behavior by kernel SHAP [44] without compromising the accuracy.

### 3.6. Overall Evaluation Criteria

To evaluate the performance of the proposed interpretable model, we have considered three stages, i.e., (a) feature importance evaluation, (b) model performance evaluation, and (c) model interpretability evaluation. For feature importance evolution from FP, the Boruta Z score is considered. The details of this calculation procedure have already been described in Section 3.2. To assess the performance of the proposed model, two measurement matrices have been considered, i.e., (a) Accuracy Score (AS) [76], and (b) Confusion Matrix (CM) [77]. The AS can be calculated by the following equation:

(26) $$AS=\left[\frac{\left(TP+TN\right)}{\left(TP+FP+TN+FN\right)}\times 100\right]\%$$

Moreover, to determine the best value of k for k-NN, a range of k-values are considered first. While considering the values within the range, only the odd values are contemplated. Therefore, if there are in total N samples present in the dataset, the range of k-values will be $\left[1,3,5,\ldots ,\sqrt{N}\right]$. Then, a grid-search approach with K fold cross validation (K-CV) is used while evaluating the performance of the classifier, whereas the values of K ranges from $\left[1,2,3,\ldots ,10\right]$. The upper range of K is arbitrary. However, the most common practice in ML is to use 10-CV by considering K = 10. Thus, the best AS matrix is decided on the best choice of k (k of k-NN) along with the best performing K-fold. Finally, the performance of the model is explained and justified by kernel SHAP.

## 4. Dataset Description 

The proposed diagnosis model is validated by two different datasets of bearings, i.e., a public dataset of bearing data provided by the Case Western Reserve University (CWRU) [46], and a rolling element bearing dataset from a self-designed testbed. Several experiments are performed to verify the efficiency and explain ability under inconsistent working conditions including different shaft speeds (r/min) and load conditions by using these two datasets.

### 4.1. Case Western Reserve University (CWRU) Bearing Dataset

**Figure 5:**
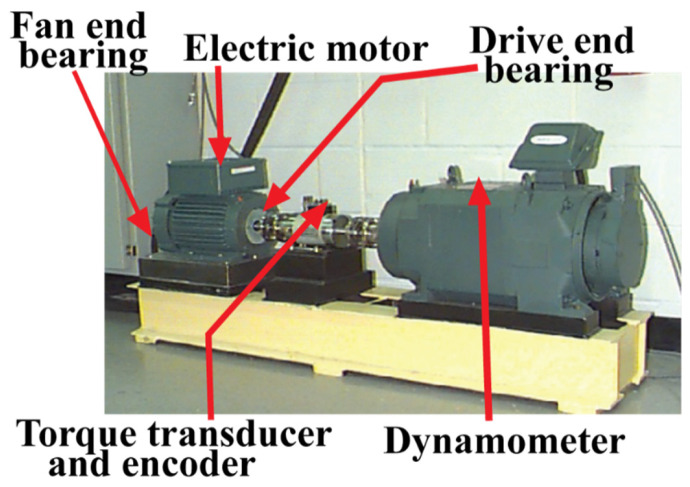
CWRU bearing testbed [46] for collecting vibration signals.

For case study 1, vibration signals of bearings are collected from a public repository provided by Case Western Reserve University [46,78]. In Figure 5, the experimental testbed is illustrated. As can be seen from this figure, the setup is composed of an induction motor of 2 horsepower, a dynamometer, and a transducer. The signals are collected from a drive end bearing with artificially seeded faults with the help of accelerometers mounted on the housing of the induction motor. With the help of the dynamometer, several motor loads were applied while recoding the signals, and as a result, variation in the motor shaft speeds was also observed, i.e., 1722–1797 revolutions per minute (r/min). The signals are collected with a sampling frequency of 12 kHz. The collected signals were associated with four types of health conditions of bearings, i.e., Normal Type (NT), Inner Raceway Type (IRT), Outer Raceway Type (ORT), and Ball Type (BT). The details of this dataset are given in Table 2. Before feeding the data to the proposed diagnostic model, we ensured that every health type has an equal number of samples, and there are no missing values in it. Thus, an ideal experimental test case is considered to analyze the performance of our algorithm.

### 4.2. Dataset from the Self-Designed Testbed

**Figure 6:**
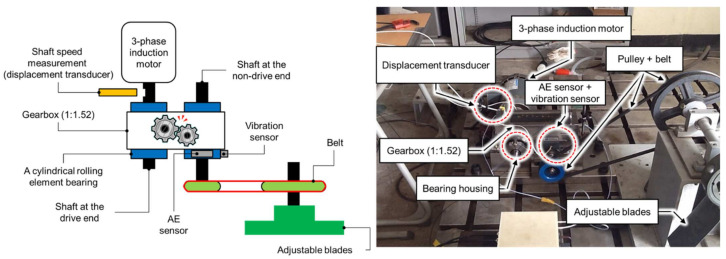
Self-designed testbed for collecting vibration signals.

**Figure 7:**
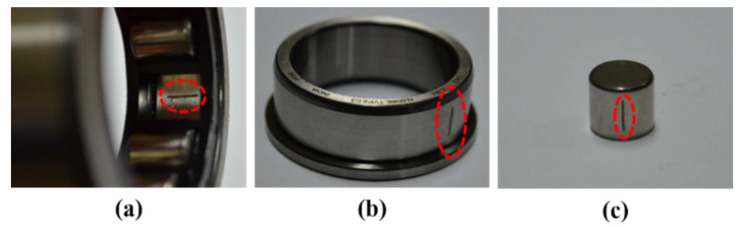
Fault types: (**a**) outer raceway type (ORT), (**b**) inner raceway type (IRT), and (**c**) roller type (RT).

Another test is conducted using vibration signals acquired from a self-designed test rig operated at three different motor speeds of 300, 400, and 500 r/min. To conduct this test, a low-speed fault simulator was designed by using a cylindrical roller bearing (FAG NJ206-ETVP2) [79]. To capture the vibration signals, a wide-band vibration sensor [76] was utilized at a sampling rate of 65,536 Hz [75]. As illustrated in Figure 6, the whole set up is composed of two shafts, i.e., a drive end shaft, and a non-drive end shaft. A gearbox with a reduction ratio of 1.52:1 was used to connect these two shafts. At the non-drive end shaft of a three-phase induction motor, a displacement transducer was enclosed to measure the shaft speed. For 0–10 kHz frequency response, this transducer has the sensitivity range of +0 to −3 dB [79]. Hence, the vibration signals were recorded under three different motor speeds i.e., 300, 400, and 500 r/min [80]. For the condition monitoring of bearing faults, three different types of seeded bearing defect were produced, as illustrated in Figure 7. For creating these artificial defects, a diamond cutter bit was used to generate cracks on the bearing surface. Therefore, the recorded signals were associated with four types of health conditions, i.e., Normal Type (NT), Inner Raceway Type (IRT), Outer Raceway Type (ORT), and Roller Type (RT). Before providing data to the model, it was ensured that every health type has an equal number of samples and that there are no missing values in it. The details of this dataset are listed in Table 3.

## 5. Experimental Result Analysis

This section presents the description of the experiments in a step-by-step manner for two separate case studies performed on two separate datasets. For every dataset, first, the performance of the classifier is analyzed for various load and speed conditions. After that, the performance score is justified based on the Kernel SHAP explanatory graph. Finally, to prove the robustness of the proposed model, the accuracy is compared with several popular state-of-the-art techniques as well.

### 5.1. Case Study 1—CWRU Dataset

**Figure 8:**
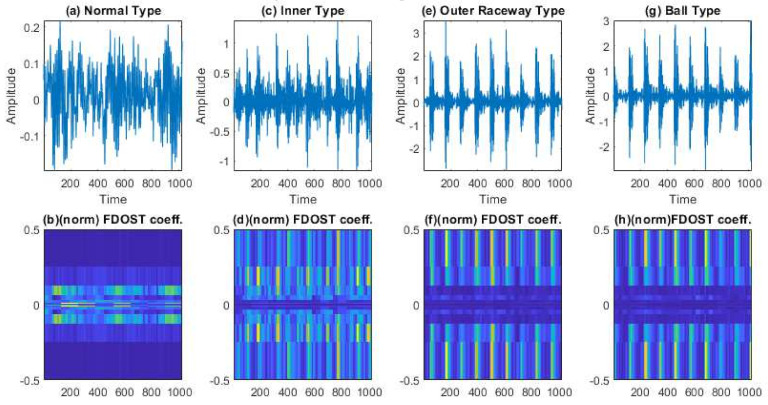
The visualization of dataset 1 in terms of raw time domain signals and respective 2D FDOST coefficient matrix—(**a**,**b**): time domain, and 2D FDOST coefficient of NT, (**c**,**d**): time domain, and 2D FDOST coefficient of IRT, (**e**,**f**): time domain, and 2D FDOST coefficient of ORT, and (**g**,**h**): time domain, and 2D FDOST coefficient of BT.

**Figure 9:**
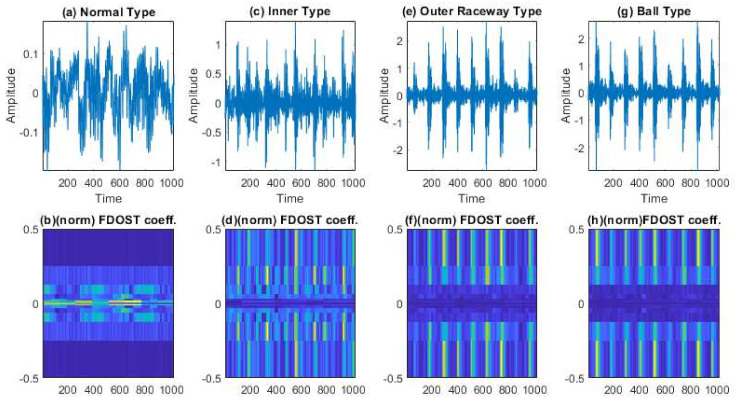
The visualization of dataset 2 in terms of raw time domain signals and respective 2D FDOST coefficient matrix—(**a**,**b**): time domain, and 2D FDOST coefficient of NT, (**c**,**d**): time domain, and 2D FDOST coefficient of IRT, (**e**,**f**): time domain, and 2D FDOST coefficient of ORT, and (**g**,**h**): time domain, and 2D FDOST coefficient of BT.

**Figure 10:**
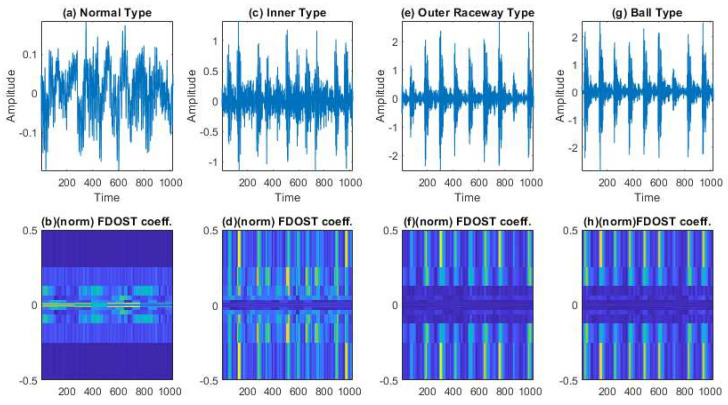
The visualization of dataset 3 in terms of raw time domain signals and respective 2D FDOST coefficient matrix—(**a**,**b**): time domain, and 2D FDOST coefficient of NT, (**c**,**d**): time domain, and 2D FDOST coefficient of IRT, (**e**,**f**): time domain, and 2D FDOST coefficient of ORT, and (**g**,**h**): time domain, and 2D FDOST coefficient of BT.

In this case, based on different load and speed conditions, three different datasets were created, which have already been described in Table 2. Each of these datasets were divided into three subsets, i.e., training, testing, and validation sets. The training and validation datasets were used for training the model with 10-CV to figure out the best model configuration with the highest performance. To generate unbiased results, model performance was calculated in terms of accuracy of the best model after 10-CV by using a totally unseen test dataset, and later, it was explained by the SHAP kernel. The train, test, and validation dataset ratios are highlighted in Table 4. First, from the raw time domain signals, the 2D FDOST coefficient matrices were calculated. As discussed earlier, this will help to preserve the time-frequency information along with the corresponding angle for both low and high frequency components of a signal. The analyzed FDOST 2D representations are given in Figure 8, Figure 9 and Figure 10 for all the datasets for visual understanding of the described phenomenon.

From Figure 8, Figure 9 and Figure 10, we can observe that time-frequency information from the FDOST is unique for each health condition. In addition, if we closely observe, we can understand that similar health conditions in all the datasets exhibit a similar type of pattern. The patterns of NT from datasets 1, 2, and 3 are very identical. Similarly, the pattern of IRT is similar for the three datasets. In the same way, it is observable and true for ORT and BT. Moreover, among the patterns of NT, IRT, ORT, and BT, there is a clear distinction, by which, after observing these patterns, we can identify the individual health type. Therefore, it can be inferred that with the suggested signal preprocessing technique, we can extract unique patterns for a given health state if underlying dataset changes or some variations within the observed dataset occur due to a change in the working condition of the machinery. This capability is mandatory to develop a bearing CM-FD model with enhanced generalization power. A bearing CM-FD model is necessary because it can identify faults if trained on one dataset and tested on a different one. Hence, there is no need to retrain the model or make changes to the overall CM-FD pipeline with a change in the application domain. However, we are not confirming this invariance before explaining the outcome of the classifier with the SHAP explanation. If we can prove that for an individual dataset, an individual model prioritizes similar types of features as important, only then we can trust a model as a reliable model to diagnose the health conditions in an invariant manner.

**Figure 11:**
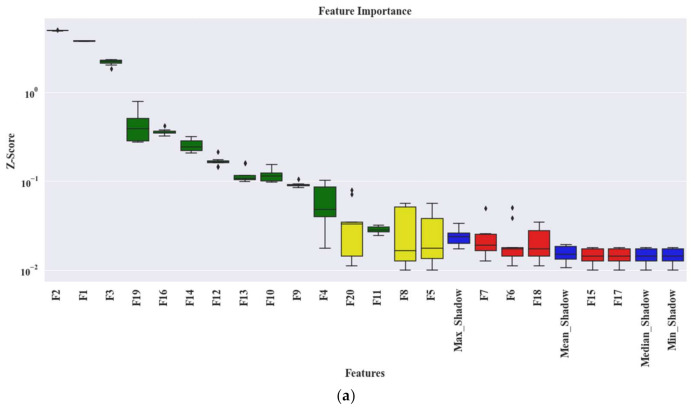

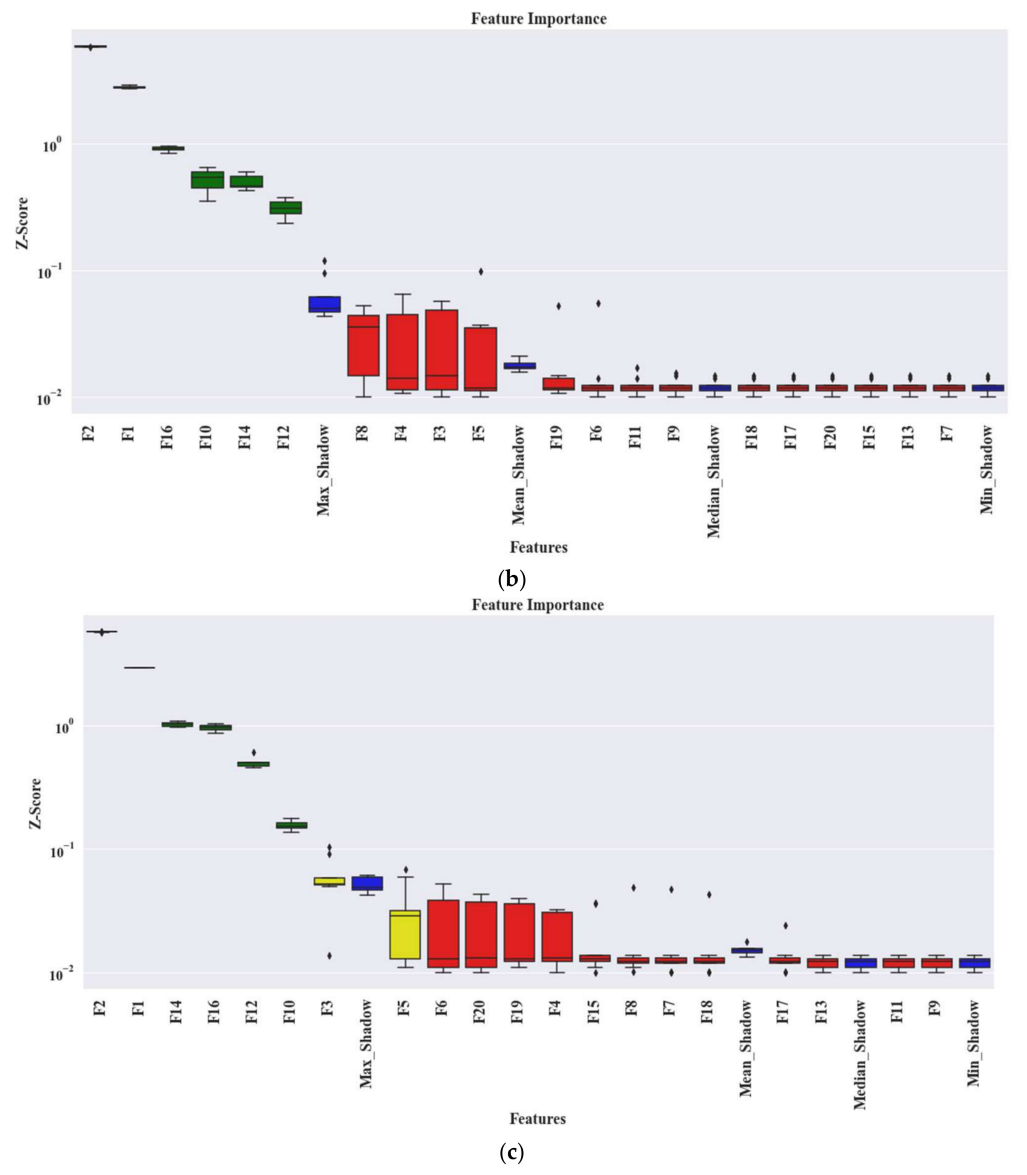
The box-whisker plot presenting Z scores generated by Boruta for each feature associated with all the datasets, i.e., (**a**) dataset 1, (**b**) dataset 2, and (**c**) dataset 3.

**Figure 12:**
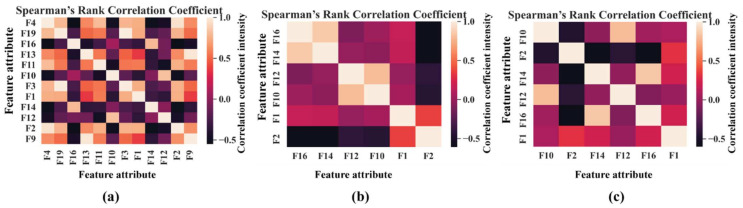
Representation of the feature correlation in the UFP of the three datasets: (**a**) UFP of dataset 1, (**b**) UFP of dataset 2, and (**c**) UFP of dataset 3.

In the next step, an SFP is formed by extracting 20 features according to the details of Table 1. Later, Boruta is applied to get the most relevant features for the given task. We are considering all the important features which are identified as important by Boruta. Intuitively, we can say that Boruta will pick only those set of feature attributes among all the extracted ones, which have class separability among different classes. Hence, to determine this phenomenon statistically, Z scores associated with each feature attributes are analyzed. Therefore, a graph with the Z scores associated with each feature are given in Figure 11 for all the datasets. From these plots, the green means the most important features, yellow means tentative features, red means unimportant/rejected features, and blue corresponds to the shadow features. Furthermore, only green features (important ones) are considered to create the UFP. Then, by removing the colinear features with a similarity score higher than 0.9 by SRCC (Figure 12), an FFP is formed, which is later provided to the final classifier to identity the faults present in the bearing dataset. With the help of FFP, only the most relevant features which directly impact the outcome of the underlying classifier can be identified. Hence, the influence of each feature on the outcome can be easily understood.

**Figure 13:**
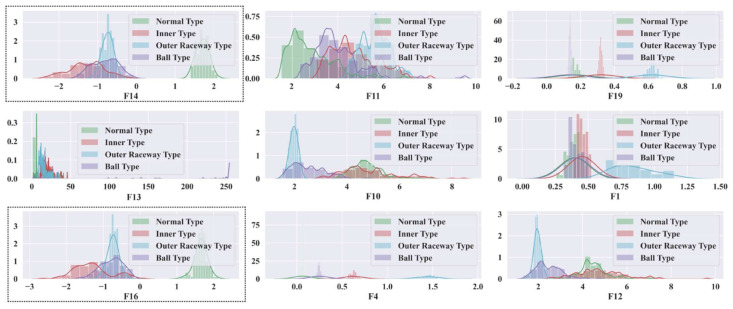
Feature distribution of FFP for model 1 in Table 5.

After forming the FFP, the training data from all the datasets are normalized to train three separate model. Each model is trained by 10-CV. With this grid search approach, the best model with the most suitable number of neighbors (k of k-NN) is picked to calculate the performance of the model in terms of accuracy. The details of these tests along with the accuracy score are highlighted in Table 5. From Table 5, we can see that for model 1, Boruta picks 12 features as the most important ones (in UFP). Then, after removing the multicollinearity by SRCC, 9 features remain as the final candidate into the feature pool (in FFP). Now, let us observe the distribution of these 9 feature attributes to examine their class separability into the following Figure 13.

**Figure 14:**
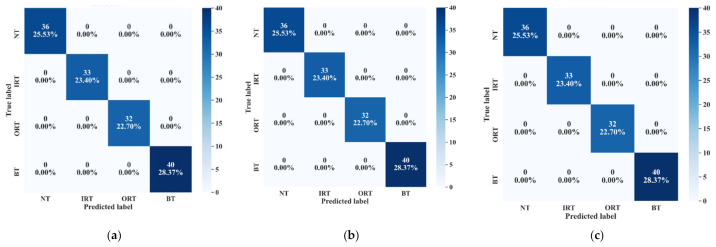
The confusion matrices of the three models that demonstrate the class-wise test accuracies, i.e., (**a**) dataset 1, (**b**) dataset 2, and (**c**) dataset3.

**Figure 15:**
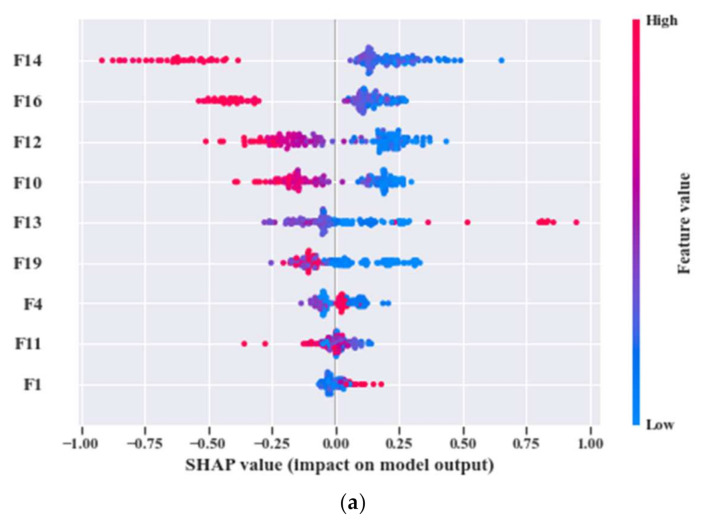

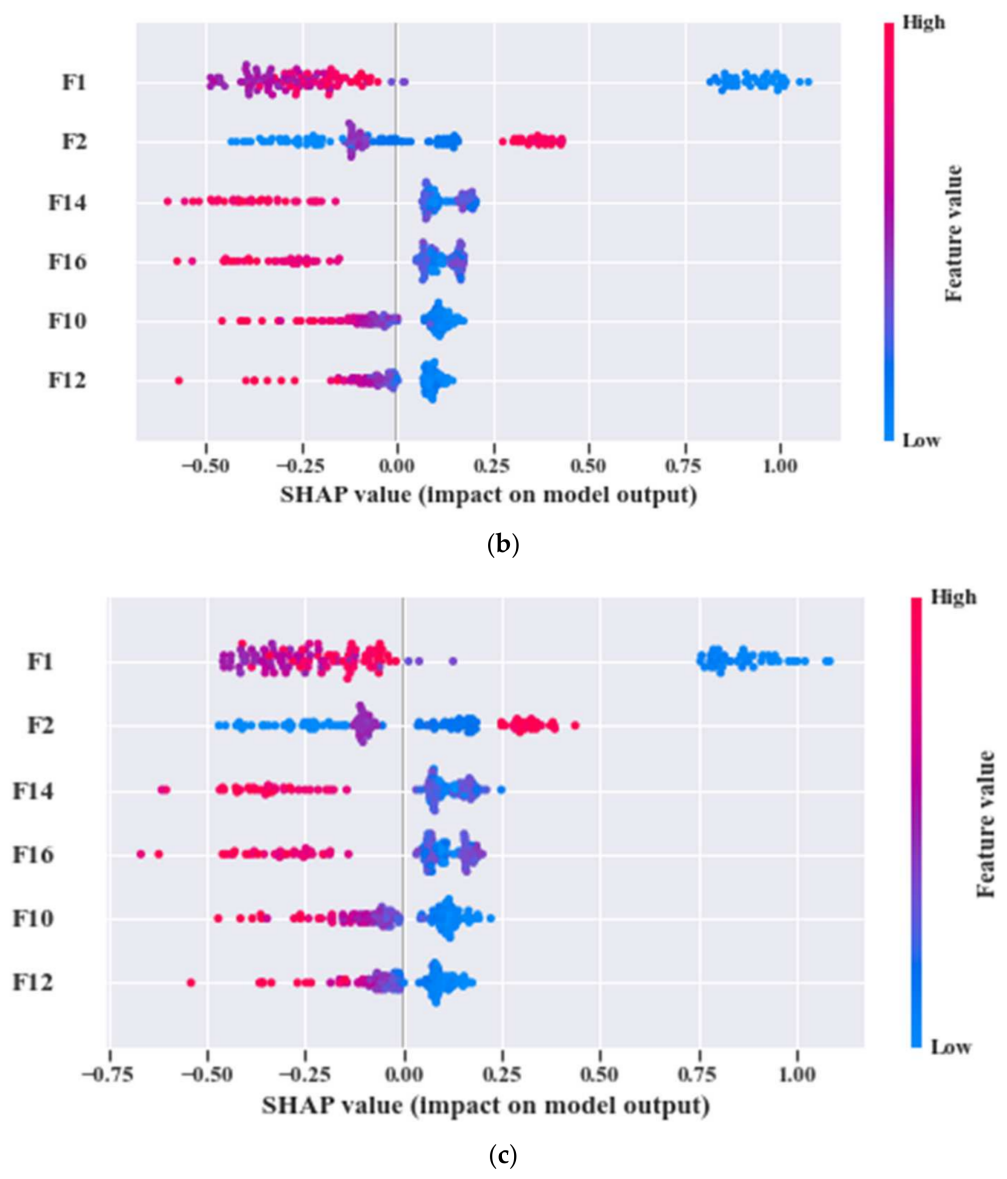
Summary plots for all the test datasets with associated SHAP values: (**a**) SHAP values for model 1, (**b**) SHAP values for model 2, and (**c**) SHAP values for model 3.

Now, by analyzing the distribution of these feature attributes, F14, and F16 can provide a very interesting insight. For both, the distribution of Normal Type (NT) is totally separate than the faulty ones. Though, the faulty health conditions have some overlap into their distributions, still there are significant difference among the Inner Raceway Type (IRT), Outer Raceway Type (ORT), and Ball Type (BT). After that, F12 and F10 have a better separability into the distribution than the others. Therefore, after passing all these selected features to the k-NN classifier, we have achieved 100% classification accuracy. However, according to our prior discussion, we need to check, for k-NN, whose feature is the most prioritized one. We analyze this part with the SHAPly additive explanations in the later portion. Before, zoom into that analysis, let us first create our own priority list of features from the distribution analysis. Based on our discussion, and observation from Figure 13, we think that the classifier will consider F14 or F16 as the first or second most important ones. After that, F12 and F10 will be prioritized. In a very similar way, the total number of selected feature attributes in UFP for both model 2 and model 3 are 9. Moreover, for these two models in UFP, there are no features which corelated more than 90% (Figure 12b–c). Therefore, no features are removed while forming the FFP through SRCC analysis. For model 1, dataset 1 is used for training as well as for testing. Similarly, for model 2—dataset 2 is used, and for model 3—dataset 3 is used. For each model, 70% of the data are used for training, and remaining 30% are used for testing. The details about the train and test split are discussed in Table 4. The confusion matrices of these three models are given in Figure 13. They demonstrate the class-wise accuracy of the models when tested with the respective datasets. From Figure 14, we can see that each model can classify all the test samples correctly from every health type, i.e., NT, IRT, ORT, and RT. That means each model achieves 100% True Positive Rate (TPR) and 100% True Negative Rate (TNR), which makes the performance of this model satisfactory with this dataset. Therefore, it can be inferred from this figure that the accuracy of each model is 100% for the individual class. In addition, in depth analysis of Table 5 reveals that in the FFP of all the three datasets, there are four common features, i.e., ‘F12’, ‘F16’, ‘F10’, ‘F14’. Therefore, it is safe to consider that these four features directly influence the final outcomes of all the three models while dealing with three different datasets. Therefore, the analysis of these common features can divulge important details about the proposed CM-FD model. This information includes: (1) in which aspect and up to which degree these common features are affecting the outcome of the model individually as well as in collectively, and (2) if the individual and collective effect of these common features is positive and of a higher degree then the model outcome can be explained with respect to these features alone. Once it is ensured that the common features have significant impact on the performance of each model, only then can a generalized CM-FD model for bearing be developed by using just these common features. A generalized CM-FD model will provide certain benefits, such as application of the same model for the bearing fault identification no matter if the underlying factors that affect the machinery operation change. Furthermore, if the generalization power of a CM-FD model is high, it can be utilized in the cross-domain applications. For this purpose, a SHAP kernel is used to explain the degree and nature of the impact these common features have on the output of each model while dealing with the three datasets. In Figure 15, the impact of each feature on the performance of each model is explained with the help of the SHAP value represented on the *x*-axis. Likewise, the *y*-axis lists the importance of the features in ascending order, where low represents the features with the least impact and high represents the features with the highest impact on the output. For instance, from our previous discussion and analysis, for model 1 in Table 5, we concluded that the classifier might consider F14 or F16 as the first or second most important ones. Therefore, from Figure 15a, it can be observed that F14 is the feature with the highest impact, then next in line is F16, then comes F12, and so on. A feature is of highest importance or relevance when its SHAP value spans over greater range than the rest, as stated in [45]. Therefore, our theoretical intuitive hypothesis is validated. Thus, we know, why a certain decision has been made, and which feature is responsible for a certain decision made by the classifier. However, our goal is to understand the impact of the four common features, i.e., ‘F12’, ‘F16’, ‘F10’, and ‘F14’ on each model’s performance. For models 2 and 3, these four features have a similar ranking. For these two models, ‘F12’, ‘F16’, ‘F10’, and ‘F14’ are the third, fourth, fifth, and sixth ranked features, respectively. Moreover, the range of their SHAP values are also similar. Similarly, for model 1, these four features are the most dominant ones, where in this case, ‘F12′ ranked ahead of ‘F10′. Another interesting observation is that for all these models, the range of SHAP values for ‘F14′ and ‘F16′ are almost similar. In the case of model 1, the range of SHAP values for these two features are even higher than those of the rest of the models. Therefore, from this analysis, it can be concluded that these common features, ‘F14′ and ‘F16′, have a noticeable contribution on the performance of all the models. Moreover, in the case of model 1, these features are the first two highly ranked features, which means that the output of the model can be supported with the help of these features. Thus, from this interpretation of the impact of common features on the output of the models with the help of the SHAP kernel [44], it is safe to consider only two features, i.e., ‘F14′ and ‘F16′, to build a generalized diagnostic model for bearing a health state assessment while dealing with different sorts of datasets. Thus, with the help of these insights, the number of task relevant features can be reduced with proper justification, which makes our model faster, robust, and easily explainable in terms of feature importance for the outcome of the model. Finally, based on the prior analysis, to evaluate the performance of the proposed generalized CM-FD model for bearing testing, from each dataset, we considered only two features, i.e., ‘F14′, and ‘F16′. To prove the robustness of this model, we performed three sperate tests. Among them, for test 1, we trained and validated (train: valid = 80:20) the model only with the samples of dataset 1, and then tested and trained the model with dataset 2 and dataset 3. Similarly, for test 2, we used dataset 2 for training and datasets 3 and 1 for testing. Likewise, for test 3, datasets 1 and 2 are used for testing, while dataset 3 is used for training the model. The details of this analysis are listed in Table 6.

All these models achieve 100% classification accuracy on the test datasets. Therefore, it can be inferred from the given results that the reduced number of features that were selected with the help of Boruta and SHAP analysis were the most relevant and important features that helped the k-NN classifier to yield maximum performance. It is noteworthy that for the first-time bearing, CM-FD is explained, and its function is interpreted with different algorithms in this manner, and this study shows the impact of such explanation on the development of a generalized CM-FD model. Additionally, to prove the robustness of the proposed bearing CM-FD model, few comparisons have been presented in Table 7 where the compared studies are conducted for a similar working environment of the machinery. The details of these comparisons are listed in Table 7. From this table, we can see that almost all the methods generate satisfactory classification accuracies for the considered scenarios. However, the compared studies have limitations in other aspects. For instance, method 1 yields 99.6% accuracy, but is unable to generate the same results if the working conditions change, i.e., variation in the motor speed and load are encountered. Moreover, another disadvantage of method 1 is that this model cannot be interpreted/explained. Thus, there is no way to debug the model for performance evaluation or adaptability for other datasets. Similarly, model 2, 3, and 4 cannot be explained and debugged. However, among them, models 2 and 4 gave 100% accuracy for datasets with variable speed and load conditions. Nevertheless, two things make our proposed model unique and state-of-the-art, i.e., (a) justification for the selection of the feature attributes, and (b) the ability of the classifier to be explained and interpreted with respect to the selected features.

### 5.2. Case Study 2—Dataset from the Self Designed Testbed

**Figure 16:**
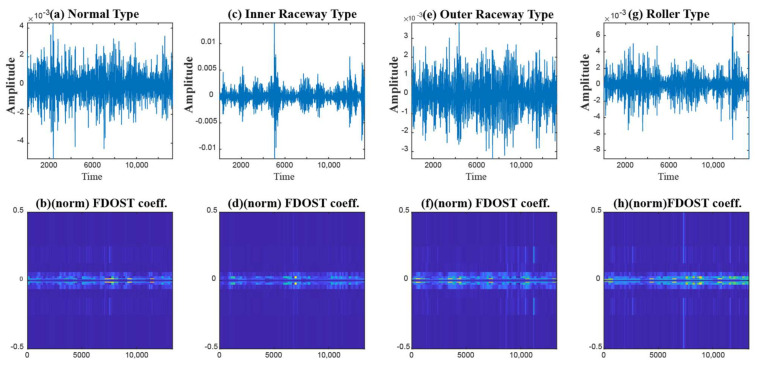
The visualization of dataset 1 in terms of raw time domain signals and the respective 2D FDOST coefficient matrix—(**a**,**b**): time domain, and 2D FDOST coefficient of NT, (**c**,**d**): time domain, and 2D FDOST coefficient of IRT, (**e**,**f**): time domain, and 2D FDOST coefficient of ORT, and (**g**,**h**): time domain, and 2D FDOST coefficient of RT.

**Figure 17:**
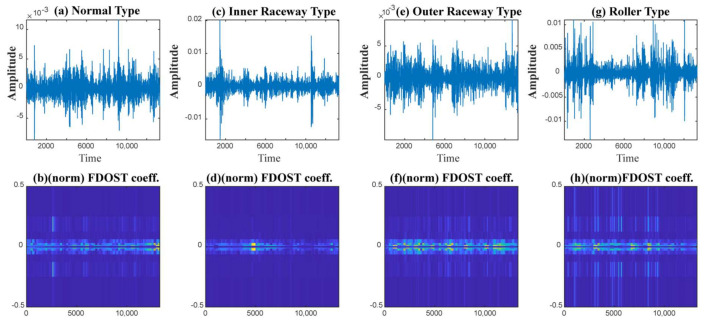
The visualization of dataset 2 in terms of raw time domain signals and the respective 2D FDOST coefficient matrix—(**a**,**b**): time domain, and 2D FDOST coefficient of NT, (**c**,**d**): time domain, and 2D FDOST coefficient of IRT, (**e**,**f**): time domain, and 2D FDOST coefficient of ORT, and (**g**,**h**): time domain, and 2D FDOST coefficient of RT.

**Figure 18:**
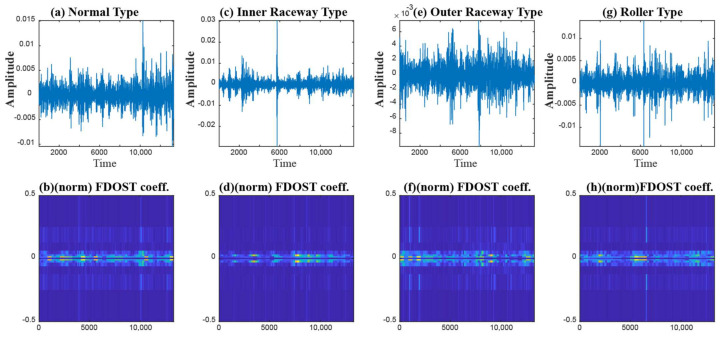
The visualization of dataset 3 in terms of raw time domain signals and the respective 2D FDOST coefficient matrix—(**a**,**b**): time domain, and 2D FDOST coefficient of NT, (**c**,**d**): time domain, and 2D FDOST coefficient of IRT, (**e**,**f**): time domain, and 2D FDOST coefficient of ORT, and (**g**,**h**): time domain, and 2D FDOST coefficient of RT.

An additional experiment was performed to confirm that the proposed model provides satisfactory results if evaluated using a totally different dataset. In this case also, like case study 1, based on different load and speed conditions, the data are divided into training, testing, and validation subsets as given in Table 8. Moreover, 10-CV is used to determine the best performing model. The test dataset remains totally unseen to the model to evaluate the model performance based on accuracy and SHAP explanations. Like the previous case study, first, from the raw time domain dataset, we calculated the FDOST 2D coefficient matrices. The 2D FDOST representations for the three datasets are given in Figure 16, Figure 17 and Figure 18 for visual understanding of the described phenomenon. From Figure 16, Figure 17 and Figure 18, we can observe that for these datasets, the time-frequency information from the FDOST is unique for each health condition. Moreover, if we closely observe, we can understand that similar health conditions from all the datasets exhibit a similar type of pattern. Although, the patterns in this case are not as vibrant as those given in Figure 8, Figure 9 and Figure 10 because the sampling frequency of this dataset is at least five times higher than the previous dataset yet are unique. Therefore, just based on the 2D FDOST analysis, it cannot be confirmed that the classifier can produce satisfactory output under variable working conditions of the machinery. Therefore, in this case, the classifier will play a vital role in the development of a generalized model by properly selecting the important features for the task. If it can be proved that for all the datasets, similar subset features have maximum influence on the output of the underlying classifier, only then can model performance be considered reliable under variable working conditions.

**Figure 19:**
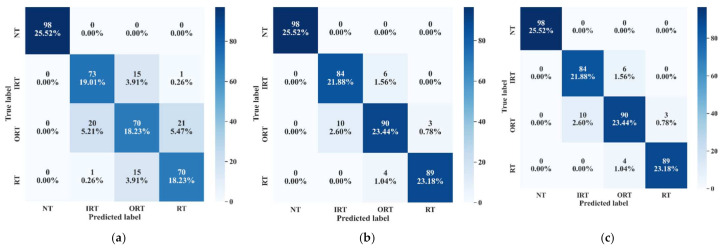
The confusion matrices of the three models that demonstrate the class-wise test accuracies, i.e., (**a**) dataset 1, (**b**) dataset 2, and (**c**) dataset 3.

In the next step, an SFP is formed by extracting 20 features according to the details given in Table 1. After that, Boruta is applied on the feature pool to select the most relevant features. The feature selection through Boruta is the same as in case study 1. Next, by removing the colinear features with a score higher than 0.9 with the help of SRCC, FFP is formed. Once the FFP is obtained, the training data from all the datasets is normalized to train three separate models. Each model is trained by 10-CV. With this grid search approach, the best model with the most suitable number of neighbors (k) is picked to measure the classification accuracy. Later, the SHAP explanation is used to figure out the common feature subset responsible for individual model performance. The details of these tests along with the accuracy scores are highlighted in Table 9. For highlighting the class-wise performance, in Figure 19, we included the confusion matrices from all the three models.

**Figure 20:**
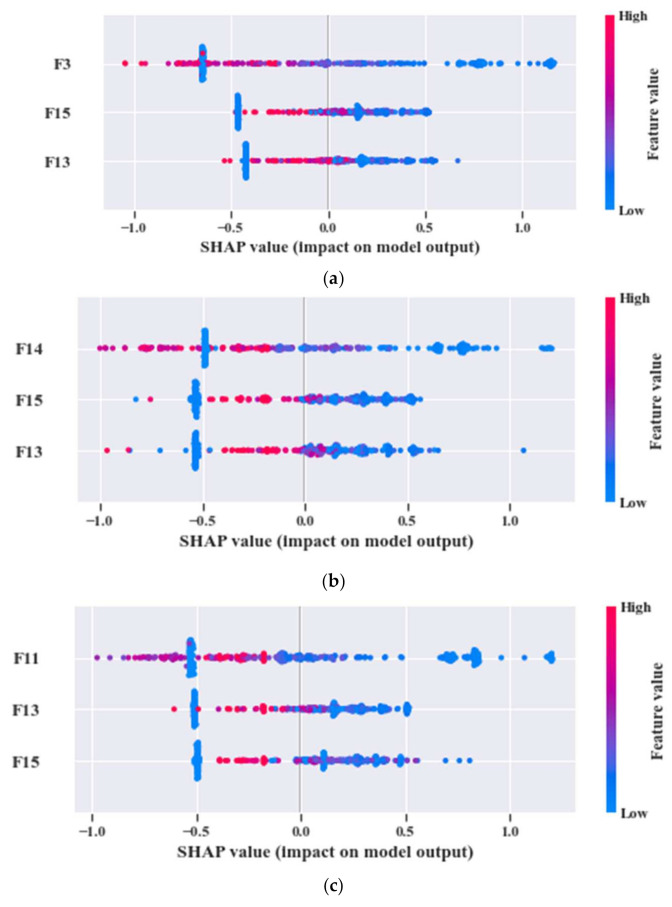
Summary plot for the test dataset on the SHAP values of (**a**) model 1, (**b**) model 2, and (**c**) model 3.

From Table 9, we can observe that ‘F13’ and ‘F15’ are the common features for the three models. However, to understand the influence of each feature and to figure out the most influential features for the k-NN classifier output, we need to observe the SHAP values of each model. In Figure 20, the given plots depict a summary of the SHAP values for the individual models. By observing all the SHAP values from Figure 20a–c, it can be inferred that ‘F13’ and ‘F15’ are highly impactful on the performance of all three models. Therefore, to justify this analysis, k-NN classifier performance is analyzed by generating diagnostic results using these two features, i.e., ‘F13’, and ‘F15’. As in the previous case study, in this case, three sperate tests were performed to evaluate the performance of the proposed model. For test 1, we train and validate (train: valid = 80:20) the model with all the samples of dataset 1, and then evaluate the performance with dataset 2 and dataset 3. Similarly, for test 2, we used dataset 2 for training, and datasets 3 and 1 for testing. Likewise, for test 3, datasets 1 and 2 are used for testing, while dataset 3 is used for training the model. All these tests achieve at least 97.0% classification accuracy on the test datasets. The details of this analysis are listed in Table 10.

## 6. Conclusions

This paper proposes an explainable AI-based approach for bearing fault diagnosis under variable speed and load conditions. A five-stage scheme is suggested to identify faults in the observed bearing signals. The first stage is data preprocessing, in which FDOST coefficient-based vibration signal preprocessing is proposed for the exploration of invariant patterns from both the time-frequency and the corresponding phase-angel information for variable speed and load conditions. Thus, the heterogeneity is first created among different health types of specific working conditions so that the signals related to each fault type are easily identifiable. The exploration of heterogenous pattern among the signals of different health types is required to improve the efficacy of subsequent steps of fault diagnosis pipeline. The next stage is feature extraction, which consists of statistical feature extraction performed on FDOST coefficients to quantify the signals from the invariant pattern of the preprocessed data. After feature extraction, an explainable feature selection process is incorporated by introducing Boruta. With the introduction of these step, the behavior of the feature selection process is being randomized to make it more robust, and bias-free. Moreover, the DT-based wrapper-based classifier helps to identify the reason for selecting certain features from the input feature set. Next, a filtration method is introduced in addition to the feature selection to avoid the multicollinearity problem. Thus, the minimum description length is obtained from the selected feature set to satisfy Occam’s Razor principal. This reduced feature set contain non-overlapping information which is helpful in generating unbiased classification results. The last stage is about the classifier interpretation, in which an additive Shapley explanation followed by k-NN is proposed to diagnose the health conditions and to explain the individual decision of the k-NN classifier for understanding and debugging the performance of the model. For the CWRU bearing dataset, our proposed approach gives 100% classification accuracy on average, and similarly, for our own experimental dataset, it gives around 97.0% classification accuracy. The conducted case studies show that the explainable model can help to build a robust classifier for invariant working scenarios with proper interpretations and explanations.

With the explainability of the feature selector and the interpretability of the classifier, we understand that for different datasets with different configurations, the proposed approach can be adopted. For adaptation of the current model for different datasets, some additional steps might be required during the feature analysis steps, i.e., changing the threshold values for discarding the colinear features, or smoothing the variables of the feature attributes to some extent. Nevertheless, having an explainable and interpretable architecture, the proposed model has better generalization capability, which can perform bearing fault diagnosis under different configurations for the variable working conditions of machines.

## Figures and Tables

**Table 1:** Feature attributes for the SFP configuration.

Input Definition	Feature Attributes				
	Mean	Std	Kurtosis	Skewness	Rms
$x=\max(A_{r,c})$	$\frac{1}{N}\overset{N}{\underset{n=1}{\sum }}x_{n}$	$\sqrt{\frac{\sum \left(x_{i}-\mu \right)}{N}}$	$\frac{1}{N}\overset{N}{\underset{n=1}{\sum }}{\left(\frac{x_{n}-\bar{x}}{\sigma }\right)}^{4}$	$\frac{1}{N}\overset{N}{\underset{n=1}{\sum }}{\left(\frac{x_{n}-\bar{x}}{\sigma }\right)}^{3}$	$\sqrt{\frac{1}{N}}\overset{N}{\underset{n=1}{\sum }}x_{n}^{2}$
$x=\max(A_{c,r})$	$\frac{1}{N}\overset{N}{\underset{n=1}{\sum }}x_{n}$	$\sqrt{\frac{\sum \left(x_{i}-\mu \right)}{N}}$	$\frac{1}{N}\overset{N}{\underset{n=1}{\sum }}{\left(\frac{x_{n}-\bar{x}}{\sigma }\right)}^{4}$	$\frac{1}{N}\overset{N}{\underset{n=1}{\sum }}{\left(\frac{x_{n}-\bar{x}}{\sigma }\right)}^{3}$	$\sqrt{\frac{1}{N}}\overset{N}{\underset{n=1}{\sum }}x_{n}^{2}$
$x=\max(\phi_{r,c})$	$\frac{1}{N}\overset{N}{\underset{n=1}{\sum }}x_{n}$	$\sqrt{\frac{\sum \left(x_{i}-\mu \right)}{N}}$	$\frac{1}{N}\overset{N}{\underset{n=1}{\sum }}{\left(\frac{x_{n}-\bar{x}}{\sigma }\right)}^{4}$	$\frac{1}{N}\overset{N}{\underset{n=1}{\sum }}{\left(\frac{x_{n}-\bar{x}}{\sigma }\right)}^{3}$	$\sqrt{\frac{1}{N}}\overset{N}{\underset{n=1}{\sum }}x_{n}^{2}$
$x=\max(\phi_{c,r})$	$\frac{1}{N}\overset{N}{\underset{n=1}{\sum }}x_{n}$	$\sqrt{\frac{\sum \left(x_{i}-\mu \right)}{N}}$	$\frac{1}{N}\overset{N}{\underset{n=1}{\sum }}{\left(\frac{x_{n}-\bar{x}}{\sigma }\right)}^{4}$	$\frac{1}{N}\overset{N}{\underset{n=1}{\sum }}{\left(\frac{x_{n}-\bar{x}}{\sigma }\right)}^{3}$	$\sqrt{\frac{1}{N}}\overset{N}{\underset{n=1}{\sum }}x_{n}^{2}$

**Table 2:** Details of the dataset collected from the CRWU bearing data bank used in case study 1.

	Health Type	Shaft Speed (r/min)	Load	Crack Size
				Length (inches)
**Dataset 1**	NT	1797	0	-
	IRT		0	0.007
	ORT		0	0.007
	BT		0	0.007
**Dataset 2**	NT	1772	1	-
	IRT		1	0.007
	ORT		1	0.007
	BT		1	0.007
**Dataset 3**	NT	1750	2	-
	IRT		2	0.007
	ORT		2	0.007
	BT		2	0.007

**Table 3:** Details of the dataset from the self-designed testbed used in case study 2.

	Health Type	Shaft Speed (r/min)	Crack Size
			Length (mm)
**Dataset 1**	NT	300	-
	IRT		6
	ORT		6
	RT		6
**Dataset 2**	NT	400	-
	IRT		6
	ORT		6
	RT		6
**Dataset 3**	NT	500	-
	IRT		6
	ORT		6
	RT		6

**Table 4:** The train, test, and validation dataset ratios.

Dataset	Train (70%)		Test (30%)	Total Samples	Sample/Health Type
	Training (80%)	Validation (20%)			
**1**	261 samples	66 samples	141 samples	468	117
**2**	261 samples	66 samples	141 samples	468	117
**3**	261 samples	66 samples	141 samples	468	117

**Table 5:** Diagnostic performance of the proposed model.

Model	Dataset	UFP	FFP	Common Feature Attributes	Best k	Accuracy (%)
**1**	1	‘F13’, ‘F4’, ‘F2’, ‘F14’, ‘F3’, ‘F11’, ‘F10’, ‘F16’, ‘F9’, ‘F12’, ‘F1’, ‘F19’	‘F13’, ‘F4’, ‘F14’, ‘F19’, ‘F11’, ‘F10’, ‘F16’, ‘F12’, ‘F1’	‘F12’, ‘F16’, ‘F10’, ‘F14’	1	100
**2**	2	‘F2’, ‘F14’, ‘F10’, ‘F16’, ‘F12’, ‘F1’	‘F2’, ‘F14’, ‘F10’, ‘F16’, ‘F12’, ‘F1’		1	100
**3**	3	‘F1’, ‘F12’, ‘F16’, ‘F10’, ‘F14’, ‘F2’	‘F1’, ‘F12’, ‘F16’, ‘F10’, ‘F14’, ‘F2’		1	100

**Table 6:** Diagnostic performance of the invariant model.

Test	Training Dataset	Test Dataset	Performance Measurement (%)—Avg.		
			TPR	TNR	Accuracy
**1**	1	2, 3	100	100	100
**2**	2	3, 1	100	100	100
**3**	3	1, 2	100	100	100

**Table 7:** Comparison analysis.

Method no.	Ref.	Signal Processing	Feature Extraction	Feature Selector	Classifier	Invariance Capability	Explain Ability	Debuggable	Accuracy (%)	Performance Gap
1	[81]	No	Time domain features: waveform length, slope sign changes, simple sign integral and Wilson amplitude inaddition to established mean absolute value and zero crossing	Laplacian Score (LS)	(Linear Discriminant Analysis) LDA, (Naïve Bayes) NB, SVM	No	No	No	99.6	0.4
2	[82]	Genetic Programming (GP)	Evolved features by GP stages	GP based filtering	k-NN	Yes	No	No	100	0.0
3	[83]	No	Local Binary Pattern (LBP)	No	Artificial Neural Network (ANN)	Yes	No	No	99.5	0.5
4	[65]	Stockwell Transform (ST)	ST coefficient imaging	No	CNN + Transfer Learning (TL)	Yes	No	No	100	0.0

**Table 8:** Details of the data division.

Dataset	Train (70%)		Test (30%)	Total Samples	Sample/Health Type
	Training (80%)	Validation (20%)			
**1**	717 samples	179 samples	384 samples	1280	320
**2**	717 samples	179 samples	384 samples	1280	320
**3**	717 samples	179 samples	384 samples	1280	320

**Table 9:** Diagnostic performance of the proposed model.

Model	Dataset	UFP	FFP	Common Feature Attributes	Best k	Accuracy (%)
**1**	1	‘F3’, ‘F13’, ‘F16’, ‘F17’, ‘F20’, ‘F9’, ‘F1’, ‘F11’, ‘F14’, ‘F7’, ‘F15’, ‘F4’, ‘F19’, ‘F12’, ‘F18’	‘F3’, ‘F13’, ‘F15’	‘F13’, ‘F15’	5	97.4
**2**	2	‘F15’, ‘F14’, ‘F19’, ‘F11’, ‘F18’, ‘F13’, ‘F9’, ‘F3’, ‘F12’, ‘F16’, ‘F20’, ‘F1’	‘F15’, ‘F14’, ‘F13’		7	97.4
**3**	3	‘F11’, ‘F19’, ‘F20’, ‘F1’, ‘F13’, ‘F15’, ‘F17’, ‘F14’, ‘F16’, ‘F3’, ‘F9’, ‘F4’	‘F11’, ‘F13’, ‘F15’		5	96.1

**Table 10:** Diagnostic performance of the invariant model.

Test	Training Dataset	Test Dataset	TPR				TNR				Accuracy (%)
			NT	IRT	ORT	RT	NT	IRT	ORT	RT	
**1**	1	2	1.00	0.93	0.95	0.98	0.99	0.99	0.97	0.99	97.0
		3	1.00	0.95	0.97	0.98	0.99	0.99	0.98	0.99	97.8
**2**	2	3	1.00	0.96	0.97	0.99	0.99	0.99	0.98	0.99	98.2
		1	1.00	0.95	0.95	0.98	0.99	0.98	0.98	0.99	97.4
**3**	3	1	1.00	0.95	0.95	0.98	0.99	0.98	0.98	0.99	97.1
		2	1.00	0.95	0.94	0.97	0.99	0.99	0.98	0.99	97.0

## Data Availability

The data of Case Study 1 are publicly available.
